# Effects of edaravone dexborneol on functional outcome and inflammatory response in patients with acute ischemic stroke

**DOI:** 10.1186/s12883-024-03712-1

**Published:** 2024-06-20

**Authors:** Wenxia Chen, Hanqing Zhang, Zhenzhen Li, Qiwen Deng, Meng Wang, Yingbin Chen, Yuan Zhang

**Affiliations:** 1 Department of Neurology, Nanjing First Hospital, Nanjing Medical University, No.68 Changle Road, Nanjing, 210006 China; 2 Department of Neurology, the Fourth Affiliated Hospital of Nanjing Medical University, No.298 Nanpu Road, Nanjing, 210000 China; 3 Department of Ultrasound Medicine, Nanjing First Hospital, Nanjing Medical University, No.68 Changle Road, Nanjing, 210006 China

**Keywords:** Acute ischemic stroke, Edaravone dexborneol, Inflammatory response, Functional outcome, Interleukin, Modified Rankin Scale

## Abstract

**Background:**

Edaravone dexborneol has been reported as an effective neuroprotective agent in the treatment of acute ischemic stroke (AIS). This study aimed at investigating the impact of edaravone dexborneol on functional outcomes and systematic inflammatory response in AIS patient.

**Methods:**

All participants were recruited from the AISRNA study (registered 21/11/2019, NCT04175691 [ClinicalTrials.gov]) between January 2022 and December 2022. The AIS patients were divided into two groups based on whether they received the treatment of edaravone dexborneol (37.5 mg/12 hours, IV) within 48 h after stroke onset. Inflammatory response was determined by detecting levels of cytokines (interleukin-2 [IL-2], IL-4, IL-5, IL-8, IL-6, IL-10, IL-12p70, IL-17, tumor necrosis factor-α [TNF-α], interferon-γ [IFN-γ], IFN-α, and IL-1β) within 14 days after stroke onset.

**Results:**

Eighty-five AIS patients were included from the AISRNA study. Patients treated with edaravone dexborneol showed a significantly higher proportion of modified Rankin Scale score < 2 compared to those who did not receive this treatment (70.7% versus 47.8%; *P* = 0.031). Furthermore, individuals receiving edaravone dexborneol injection exhibited lower expression levels of interleukin (IL)-1β, IL-6, and IL-17, along with higher levels of IL-4 and IL-10 expression during the acute phase of ischemic stroke (*P* < 0.05). These trends were not observed for IL-2, IL-5, IL-8, IL-12p70, tumor necrosis factor-α, interferon-γ [IFN-γ], and IFN-α (*P* > 0.05).

**Conclusions:**

Treatment with edaravone dexborneol resulted in a favorable functional outcome at 90 days post-stroke onset when compared to patients without this intervention; it also suppressed proinflammatory factors expression while increasing anti-inflammatory factors levels.

**Trial registration:**

ClinicalTrials.gov NCT04175691. Registered November 21, 2019, https://www.clinicaltrials.gov/ct2/show/NCT04175691.

## Background

Stroke is a major cause of acquired adult disability and mortality worldwide [1]. Intravenous thrombolysis and mechanical thrombectomy are currently the two most efficacious therapeutic approaches for acute ischemic stroke (AIS). Despite significant advancements in reperfusion treatment for ischemic stroke, the rate of clinically ineffective reperfusion remains around 50% in AIS patients [2]. Moreover, numerous clinical trials investigating neuroprotective agents have failed to demonstrate any clinical benefits [3]. The SAINT I and II trials suggested that neuroprotective agent NXY-059 was ineffective for AIS treatment within 6 h after stroke onset [4]. Nerinetide was demonstrated to fail to improve functional outcomes after endovascular therapy [5]. The ALIAS trials also showed that 25% albumin (2 g/kg, IV) did not improve 90-day clinical outcomes and increased the incidence of pulmonary edema and intracerebral hemorrhage [6]. Another neuroprotective agent (magnesium sulfate) within 2 h after stroke onset also failed to improve functional outcomes at 90 days [7]. Therefore, there is an urgent need to identify an effective neuroprotective agent that can reduce disability and mortality rates in AIS management.

A novel neuroprotective agent of Edaravone dexborneol could protect against ischemic damage by multifunctional cytoprotective pathways including inflammatory, excitotoxic, oxidative and apoptotic insults [8]. The Treatment of Acute Ischemic Stroke with Edaravone Dexborneol (TASTE) trial, a phase III, randomized, double-blind, parallel, comparative study, enrolled 1200 participants with AIS, which has reported an effective neuroprotective agent of edaravone dexborneol in the improvement of 90-day functional outcomes [9]. Furthermore, the following TASTE-SL trial also showed sublingual edaravone dexborneol achieved a favorable outcome at 90 days in patients with AIS within 48 h [10]. Previous studies have highlighted its involvement in inhibiting pro-inflammatory factors and enhancing blood-brain barrier permeability following ischemic stroke [11, 12]. Additionally, edaravone dexborneol promotes microglial activation towards the M2 phenotype by modulating aryl hydrocarbon receptor expression, thereby exerting anti-inflammatory effects [13]. Therefore, edaravone dexborneol regulated inflammatory response to exert a neuroprotective effect. However, the systemic inflammatory factors (interleukin-2 [IL-2], IL-4, IL-5, IL-8, IL-6, IL-10, IL-12p70, IL-17, tumor necrosis factor-α [TNF-α], interferon-γ [IFN-γ], IFN-α, and IL-1β) associated with edaravone dexborneol treatment in AIS management remains unclear.

In this study, we aimed to investigate the expression profile of systemic inflammatory factors and clinical benefits following edaravone dexborneol treatment in the acute phase of AIS patients.

## Materials and methods

### Study population

The informed consent was obtained from all individuals. The study protocol adhered to the Declaration of Helsinki and received approval from the Ethics Committee of Nanjing First Hospital, Nanjing Medical University. A total of 85 patients with AIS were prospectively enrolled in an observational study of the AISRNA study (www.clinicaltrials.gov, NCT04175691). All participants were recruited from the Department of Neurology at Nanjing First Hospital, Nanjing Medical University. Inclusion criteria for enrollment included: [1] confirmation of anterior circulation cerebral infarction through non-contrast computer tomography (NCCT) or magnetic resonance imaging (MRI); [2] treatment initiation with edaravone dexborneol within 48 h after stroke onset, or absence of treatment with edaravone dexborneol; [3] age between 18 and 80 years old; and [4] National Institute of Health Stroke Scale (NIHSS) score ranging from 4 to 24. Exclusion criteria consisted of: [1] received the treatment with intravenous thrombolysis or mechanical thrombectomy after stroke onset; [2] presence of infectious diseases upon admission; [3] modified Rankin Scale score > 2 before stroke onset; [4] immunosuppressive therapy or antibiotic treatment within the past four weeks; [5] concurrent malignant tumors or severe organ failure including kidney, liver, and heart failure; [6] dysphagia; [7] lack of informed consent.

### Procedures

All patients underwent standardized treatment according to AIS guidelines [14]. The standardized group included antiplatelet aggregation or anticoagulant therapy, statin therapy, and control of risk factors regarding AIS. The treatment group received intravenous infusion of edaravone dexborneol at a dose of 37.5 mg administered by neurological nurses every 12 h for 14 days or hospital discharge and the standardized treatment.

### Biomarkers of inflammatory response

Whole blood samples were collected upon admission and subsequently on day 2–3, 5–7, and 10–14. Plasma samples were then extracted and stored at -80℃. The concentrations of various inflammatory factors (IL-2, IL-4, IL-5, IL-8, IL-6, IL-10, IL-12p70, IL-17, TNF-α, IFN-γ, IFN-α, and IL-1β) were systematically measured by the 12-Cytokine Detection Kit (Raisecare, Qingdao, China) and Navios flow cytometer (Beckman Coulter, California, USA) following the manufacturer’s protocol.

### Clinical characteristics

Demographics, medical history, stroke severity evaluation by NIHSS score assessment criteria [15] as well as stroke etiology based on Trial of Org 10,172 in acute stroke treatment (TOAST) criteria [16] were prospectively collected. Functional outcomes were assessed by modified Rankin Scale [17]. Table 1 provides detailed baseline characteristics of patients with AIS.

**Table 1:** Baseline characteristics of the enrolled patients

Variable	Total (*n* = 85)	Treatment group (*n* = 41)	Standardized group (*n* = 44)	*p*-value
Demographics				
Male, n (%)	67 (65.9)	26 (63.4)	30 (68.2)	0.643
Age (years)	67.27 ± 11.30	65.15 ± 10.82	68.06 ± 11.55	0.235
Medical history, n (%)				
Hypertension	57 (67.1)	27 (65.9)	30 (68.2)	0.819
DM	18 (21.2)	9 (22.0)	9 (20.5)	0.866
CAD	14 (16.5)	6 (14.6)	8 (18.2)	0.659
AF	18 (21.2)	8 (19.5)	10 (22.7)	0.717
IS or TIA	13 (15.3)	6 (14.6)	7 (15.9)	0.623
Stroke etiology^a^, n (%)				
LAA	51 (60.0)	24 (58.5)	27 (61.4)	0.957
Cardioembolism	6 (7.1)	3 (7.3)	3 (6.8)	
SAO	23 (27.1)	12 (29.3)	11 (25.0)	
Other	5 (5.9)	2 (4.9)	3 (6.8)	
NIHSS score on admission	5 (4–10)	5 (4–9)	5 (4–10)	0.826
mRS score at admission	3 (1–4)	3 (2–4)	3 (1–4)	0.147
Laboratory characteristics				
hs-CRP (µg/mL), median (IQR)	5.00 (4.46–11.62)	5.00 (4.79–12.93)	5.00 (3.88–11.32)	0.729
WBC (10^9^/L)	10.17 ± 3.04	10.09 ± 3.16	10.23 ± 2.96	0.834
Creatinine (µmol/L), median (IQR)	67.0 (58.0-80.6)	71.0 (58.8–82.6)	66.2 (57.3–75.0)	0.289
LDL-C (mmol/L)	2.64 ± 1.01	2.74 ± 0.93	2.56 ± 1.08	0.395

DM, diabetes mellitus; CAD, coronary artery disease; AF, atrial fibrillation; IS, ischemic stroke; TIA, transient ischemic attack; IQR, interquartile range; NIHSS, National Institute of Health Stroke Scale; LAA, large artery atherosclerosis; SAO, small artery occlusion; hs-CRP, high-sensitivity C-reactive protein; LDL-C, low-density lipoprotein cholesterol; mRS, modified Rankin scale

Treatment group received edaravone dexborneol intravenous infusion of 37.5 mg/dose (once every 12 h) on the basis of the standardized group

Standardized group received antiplatelet aggregation or anticoagulant therapy, statin therapy, and control of risk factors regarding ischemic stroke

Continuous variables are expressed as median (interquartile range) or mean ± standard deviation (SD). Categorical variables are expressed as frequency and percentage

^a^ According to the modified TOAST classification

### Statistical analysis

Baseline characteristics between the groups were compared by using the SPSS 20.0 system. Continuous variables including inflammatory factors are expressed as the mean ± standard deviation (SD) or median (interquartile range [IQR]), and comparisons were made using t-test or one-way ANOVA if applicable; otherwise, Mann–Whitney U test was used. Categorical variables are presented as frequency (percentage) and compared using chi-square test. A *P* value less than 0.05 was considered statistically significant.

## Results

### Baseline characteristics

**Fig. 1:**
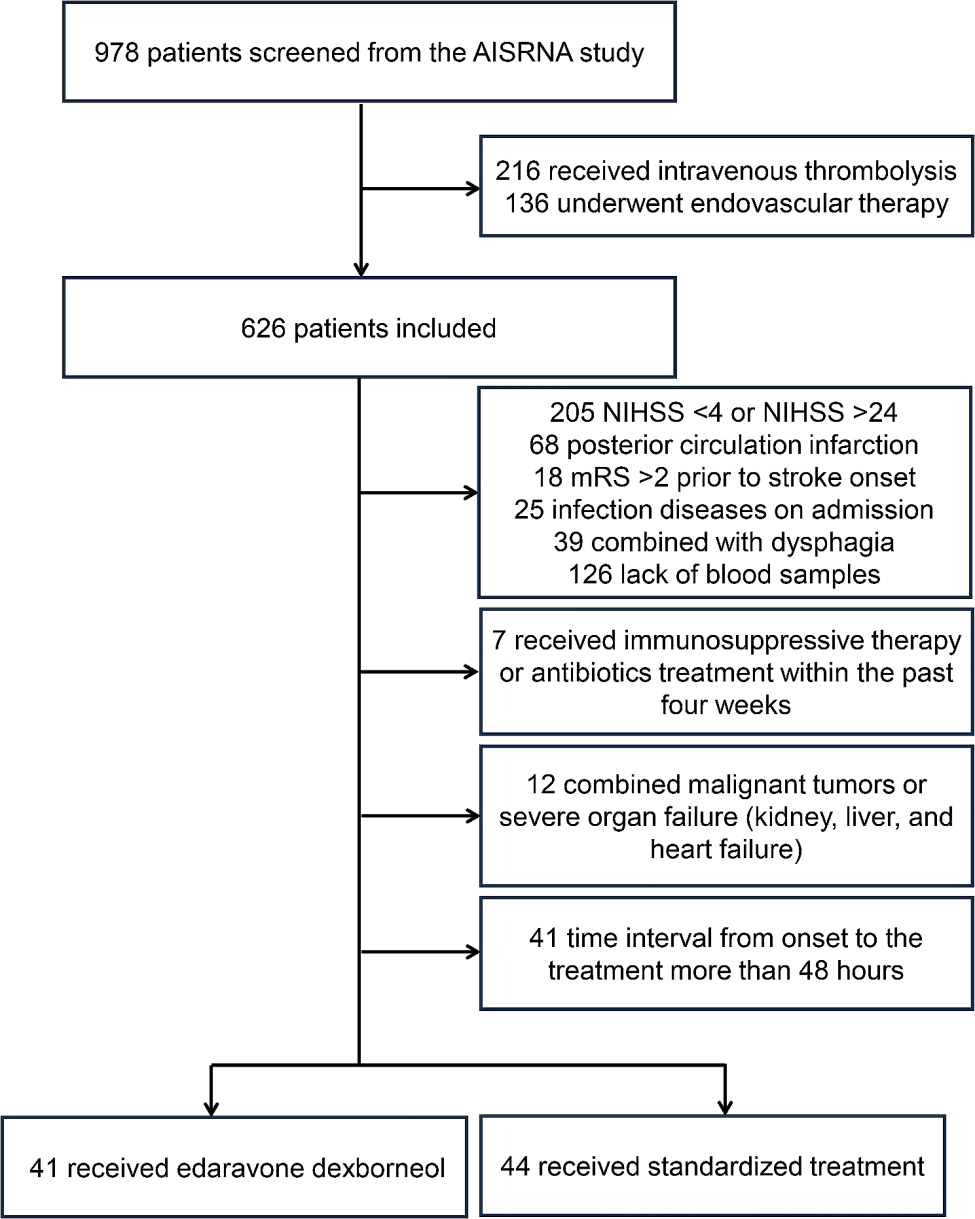
Flowchart of the enrolled patients NIHSS, National Institute of Health Stroke Scale; mRS, modified Rankin Scale

Among the 978 AIS patients screened from the AISRNA study between January 2022 and December 2022, a total of 893 patients were excluded. The primary exclusions included: intravenous thrombolysis (*n* = 216), endovascular therapy (*n* = 136), NIHSS scores < 4 or > 24 (*n* = 205), posterior circulation infarction (*n* = 68), and invalid blood samples (*n* = 126). Therefore, 85 patients (44 patients received the standardized treatment [standardized group] and 41 patients received edaravone dexborneol plus standardized treatment [treatment group]) were included in the final analysis (Fig. 1). No significant differences in baseline characteristics were observed between these two groups (Table 1).

### Association of edaravone dexborneol with clinical outcomes

**Fig. 2:**
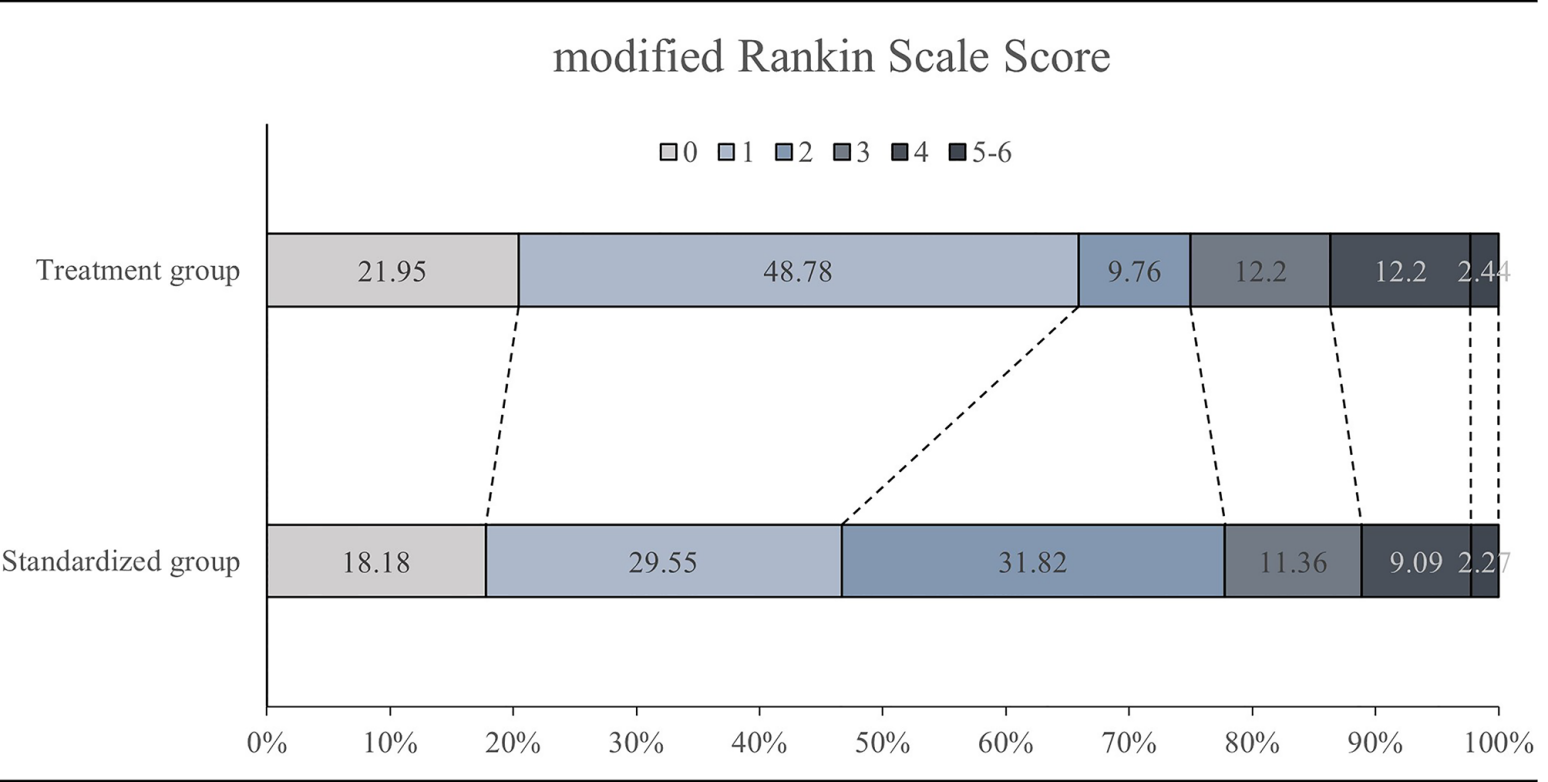
Distribution of the 90-day modified Rank Scale scores

There was no significant difference in NIHSS scores on admission between the treatment group and standardized group (*P* = 0.826). However, the treatment group exhibited lower NIHSS scores compared to the standardized group on day 10–14 (*P* = 0.019). There were no significant differences in NIHSS scores on day 2–3 and day 5–7 between these two groups (*P* > 0.05, Table 2). Additionally, there were 29 (70.7%) patients in the treatment group and 21 (47.8%) patients in the standardized group with mRS score < 2 on day 90 (*P* = 0.031, Table 2; Fig. 2). However, we did not observe a significant difference between the treatment (*n* = 33, 80.5%) and standardized groups (*n* = 35, 79.5%) with mRS score ≤ 2 on day 90 (*P* = 0.914, Fig. 2).

**Table 2:** Association of edaravone dexborneol on clinical outcomes in patients with ischemic stroke

Variable	Total (*n* = 85)	Treatment group (*n* = 41)	Standardized group (*n* = 44)	*p*-value
NIHSS on D2-3, median (IQR)	5 (3–8)	4 (3–6)	5 (2–10)	0.507
NIHSS on D 5–7, median (IQR)	5 (2–8)	4 (2–6)	5 (2–10)	0.472
NIHSS on D10-14, median (IQR)	3 (1–7)	2 (1–4)	4 (2–8)	0.019
mRS<2 on D90, n (%)	50 (58.8)	29 (70.7)	21 (47.8)	0.031

NIHSS, National Institute of Health Stroke Scale; IQR, interquartile range; mRS, modified Rankin scale

D2-3 indicates day 2–3 after stroke onset; D5-7, day 5–7; D10-14, day 10–14; D90, day 90

Treatment group received edaravone dexborneol intravenous infusion of 37.5 mg/dose (once every 12 h) on the basis of the standardized group

Standardized group received antiplatelet aggregation or anticoagulant therapy, statin therapy, and control of risk factors regarding ischemic stroke

### Dynamic change of inflammatory factors after treatment

**Fig. 3:**
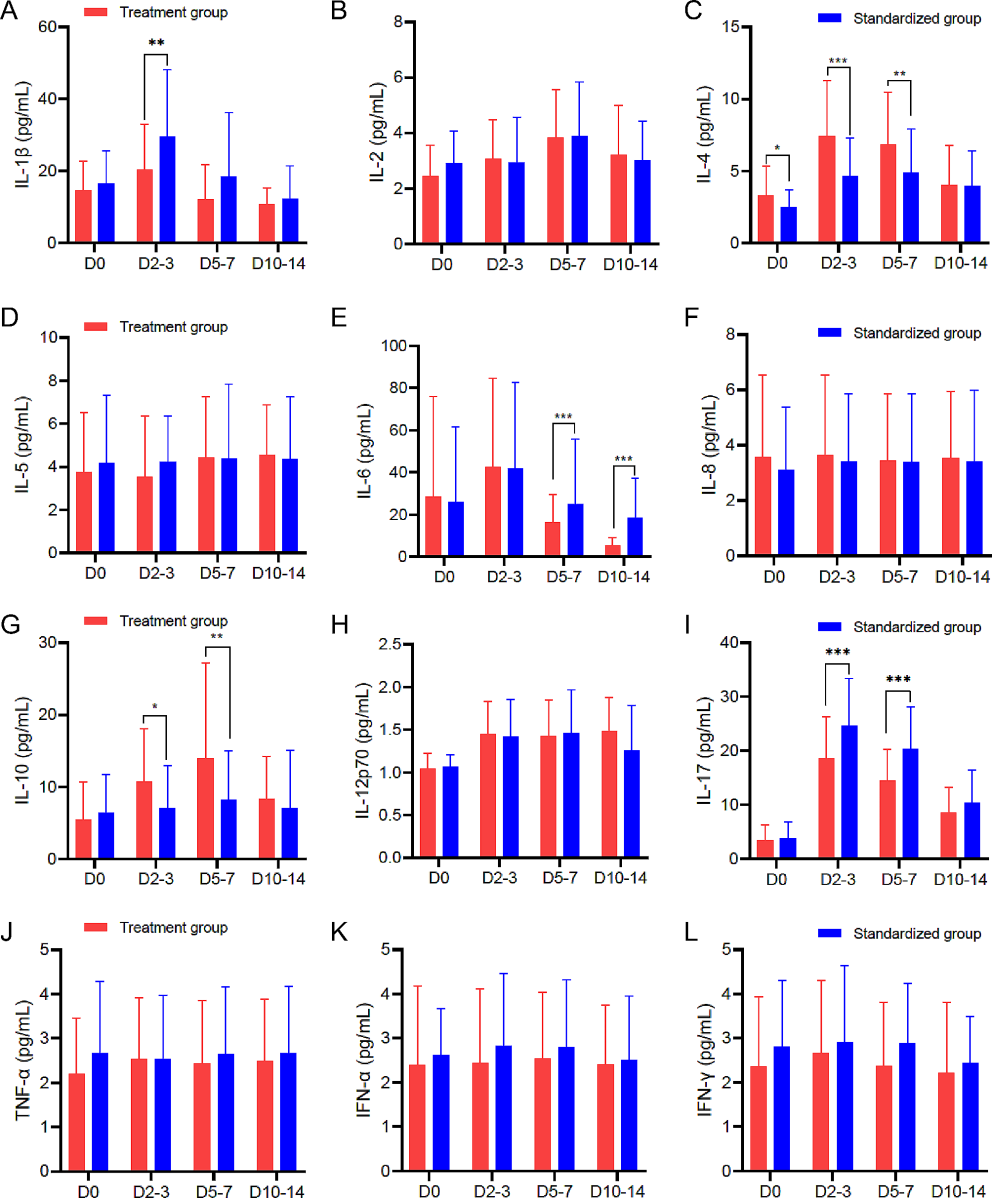
Dynamic change of inflammatory factors after admission in patients with ischemic stroke Compared to the standard group, the treatment group had lower levels of IL-1β on day 2–3, IL-6 on day 5–7 and day 10–14, and IL-17 on both day 2–3 and day 5–7 (A, E, and I), as well as higher levels of IL-4 on day 2–3 and day 5–7 and higher levels of IL-10 on day 2–3 and day 5–7 (C and G). Other inflammatory factors were no statistical differences between the two groups (B, D, F, H, J, K, and L). IL, interleukin; TNF, tumor necrosis factor; IFN, interferon-γ; D, day. *P < 0.05, *P < 0.01, *P < 0.001

Given the role of inflammatory response in acute ischemic stroke [18, 19], we explored the influence of edaravone dexborneol on inflammatory response during the acute phase of ischemic stroke. We collected four samples at different time points: admission, day 2–3, day 5–7, and day 10–14. The results showed no significant differences in 11 inflammatory factors between the two groups upon admission except for IL-4 (*P* > 0.05, Table 3). However, compared to the standard group, the treatment group had lower levels of IL-1β and IL-17 on day 2–3, and higher levels of IL-4 and IL-10 on day 2–3 (*P* < 0.05, Fig. 3A, C, G, and I). On day 5–7, high levels of IL-4 and IL-10 were observed in the treatment group (Fig. 3C and G), but IL-6 and IL-17 exhibited an opposite effect in the treatment group (Fig. 3E and I). On day 10–14, we only found decreased levels of IL-6 in the treatment group (*P* < 0.001, Fig. 3E).

**Table 3:** Baseline levels of inflammatory factors in patients with ischemic stroke

Variable	Total (*n* = 85)	Treatment group (*n* = 41)	Standardized group (*n* = 44)	*p*-value
IL-1β (pg/mL)	15.58 ± 8.99	14.61 ± 8.25	16.51 ± 9.14	0.314
IL-2 (pg/mL)	2.68 ± 1.17	2.44 ± 1.13	2.90 ± 1.18	0.073
IL-4 (pg/mL)	2.86 ± 1.72	3.28 ± 2.04	2.46 ± 1.26	0.028
IL-5 (pg/mL)	3.95 ± 2.97	3.74 ± 2.77	4.15 ± 3.17	0.537
IL-6 (pg/mL)	27.21 ± 41.47	28.59 ± 47.30	25.93 ± 35.70	0.769
IL-8 (pg/mL)	3.32 ± 2.62	3.56 ± 2.97	3.10 ± 2.27	0.420
IL-10 (pg/mL)	5.90 ± 5.31	5.44 ± 5.20	6.33 ± 5.43	0.445
IL-12p70 (pg/mL)	1.05 ± 0.16	1.04 ± 0.18	1.07 ± 0.14	0.489
IL-17 (pg/mL)	3.72 ± 2.85	3.53 ± 2.78	3.89 ± 2.93	0.565
TNF-α (pg/mL)	2.44 ± 1.47	2.20 ± 1.25	2.67 ± 1.62	0.138
IFN-α(pg/mL)	2.51 ± 1.45	2.39 ± 1.79	2.62 ± 1.05	0.464
IFN-γ (pg/mL)	2.59 ± 1.54	2.36 ± 1.57	2.81 ± 1.50	0.180

IL, interleukin; TNF, tumor necrosis factor; IFN, interferon

Treatment group received edaravone dexborneol intravenous infusion of 37.5 mg/dose (once every 12 h) on the basis of the standardized group

Standardized group received antiplatelet aggregation or anticoagulant therapy, statin therapy, and control of risk factors regarding ischemic stroke

## Discussion

The present study of AIS patients from the AISRNA study demonstrated an improvement role of edaravone dexborneol on functional outcome in acute ischemic stroke. Furthermore, edaravone dexborneol inhibited proinflammatory factors expression and increased anti-inflammatory factors levels during the acute phase of ischemic stroke.

Over the past ten years, a series of clinical studies focusing on neuroprotection have failed to show significant benefits for ischemic stroke [4, 7, 20–22]. However, a phase III clinical trial has shown that administering edaravone dexborneol within 48 h after stroke onset leads to improved functional outcomes at day 90 compared to edaravone alone [9]. Ischemic stroke results in damage through various pathways in brain ischemia. Previous drugs targeting neuroprotection only interfere with a single mechanism of brain damage. In contrast, edaravone dexborneol protected against ischemic injury by multifunctional cytoprotective pathways including inflammatory, excitotoxic, oxidative and apoptotic insults [8, 9, 23]. This study also demonstrates that edaravone dexborneol improves functional outcomes at day 90 in patients with acute ischemic stroke.

Poststroke inflammatory response is involved in brain injury [24]. Our previous study has shown that inflammatory factors have predictive value for stroke progression in patients undergoing endovascular therapy [25]. Edaravone has been reported to protect endothelial and neuronal cells during brain ischemia through suppressing inflammation and neurotoxicity [26, 27]. Bornel shows potential as a neuroprotective agent by suppressing reactive oxygen species generation and inhibiting adverse inflammatory responses while reducing NO and NO synthase levels [28]. A series of clinical and animal studies have shown that edaravone dexborneol alleviated ischemic brain damage by multiple molecular mechanisms including its effect on inflammatory response [8, 12, 29–32]. Additionally, a previous study showed that edaravone dexborneol reduced the levels of pro-inflammatory cytokines (IL-1β, IL-6 and TNF-α) in the APP/PS1 mice [33]. Importantly, edaravone dexborneol also suppressed the production of IL-1β, IL-6 and TNF-α and inhibited macrophages polarization to alleviate ischemic injury [34]. Another study demonstrated that edaravone dexborneol facilitated M2 polarization of microglia through inhibiting TL4/NF-κB pathway [35]. Furthermore, edaravone dexborneol exerted neuroprotective effect by inhibiting NF-κB/NLRP3/GSDMD signaling pathway and inflammatory factors (IL-1β and IL-18) in experimental ischemic stroke [32]. These studies suggested that edaravone dexborneol may suppress inflammatory response through regulating macrophages polarization and NLRP3 inflammasome. Our study demonstrated that edaravone dexborneol effectively inhibited proinflammatory factors and increased anti-inflammatory factors during the acute phase of ischemic stroke.

The strengths of our study include the exclusion of interference from intravenous thrombolysis and endovascular therapy on inflammatory response, as well as an opposite effect of edaravone dexborneol on proinflammatory and anti-inflammatory factors during acute phase of ischemic stroke. There are several limitations to acknowledge. Firstly, this study was an observational and single center. Secondly, whole blood samples were collected upon admission, on day 2–3, 5–7, and 10–14. Thus, blood sampling schedule was irregular. Thirdly, we only observed dynamic changes in inflammatory factors during the acute phase without exploring further potential mechanisms underlying the suppression of inflammatory response by edaravone dexborneol. Lastly, some patients received treatment approximately 48 h after stroke onset which may have influenced the levels of inflammatory factors on day 2–3 or day 5–7.

## Conclusions

This study demonstrated that the 90-day good functional outcome favored patients treated with edaravone dexborneol who had lower levels of proinflammatory factors and higher levels of anti-inflammatory factors compared to patients without this treatment.

## Data Availability

All data supporting our findings are available from the corresponding authors upon reasonable.

## Abbreviations

AIS: acute ischemic stroke
modified Rankin Scale: mRS
NCCT: non-contrast computer tomography
MRI: magnetic resonance imaging
IL: interleukin-2
TNF: tumor necrosis factor-α
IFN: interferon-γ
